# Planning impairment in schizophrenia: The possible role of abstract thinking and short-term memory

**DOI:** 10.1192/j.eurpsy.2021.1404

**Published:** 2021-08-13

**Authors:** A. Alekseev, M. Morozova, G. Rupchev

**Affiliations:** Laboratory Of Psychopharmacology, FSBSI Mental Health Research Center, Moscow, Russian Federation

**Keywords:** Planning, schizophrénia, Abstract thinking, short-term memory.

## Abstract

**Introduction:**

The planning impairment is one of the basic aspect of cognitive dysfunction, but its mechanisms in schizophrenia remain unclear.

**Objectives:**

To assess the links between planning and cognitive functioning in schizophrenic patients and in norm.

**Methods:**

50 patients with schizophrenia (age 34.92±8.54; illness duration 8.34±5.87) and 50 healthy volunteers (age 32.42±7.26) were examined. Brief Assessment of Cognition in Schizophrenia, Benton’s test for short-term memory assessment; sub-test Similarity (from WAIS) to assess abstract thinking were used.

**Results:**

Patients showed significantly worse results in all parameters (Tab.1). Table 1: Differences of planning between groups.

**Table:** 

	Schizophrenia	Norm	p-level
TOL-DX	92,64±14,48	102,52±11,97	0,00033
Similarity	16,92±3,97	19,76±2,85	0,00009
BVTR Score	6,73±1,78	7,60±1,32	0,00709

In healthy subjects, significant relationship was found between planning and abstract thinking, and there was no relationship between planning and short-term memory (Tab.2). Table 2: Correlations in the Norm group

**Table:** 

	Spearman R	p-level
TOL-DX & Similarity	0,392530	0,004809
TOL-DX & BVTR	0,186494	0,194710

In patients with schizophrenia, the opposite picture was observed (Tab.3). Table 3: Correlations in the Schizophrenia group.

**Table:** 

	Spearman R	p-level
TOL-DX & Similarity	0,262389	0,071596
TOL-DX & BVTR	0,344566	0,015331

The effectiveness of planning in patients was significantly associated with short-term memory, but not with abstract thinking.

**Conclusions:**

Study results indicate a possible role of basic aspects of mental activity such as short-term memory in planning impairment in patients with schizophrenia. Problem solving and reasoning disorders represent two relatively independent forms of thought disorders in schizophrenia.

